# *Staphylococcus aureus* bacteraemia, cardiac implantable electronic device, and the risk of endocarditis: a retrospective population–based cohort study

**DOI:** 10.1007/s10096-023-04585-x

**Published:** 2023-03-15

**Authors:** Andreas Berge, Casper Carlsén, Alexandros Petropoulos, Fredrik Gadler, Magnus Rasmussen

**Affiliations:** 1 Unit of Infectious Diseases, Department of Medicine, SolnaKarolinska Institutet, 17176 Stockholm, Sweden; 2 Department of Infectious Diseases, Karolinska University Hospital, Stockholm, Sweden; 3 Department of Microbiology, Tumor and Cell Biology, Karolinska Institutet, Stockholm, Sweden; 4 Department of Clinical Microbiology, Karolinska University Hospital, Stockholm, Sweden; 5 Unit of Cardiology, Department of Medicine, SolnaKarolinska Institutet, Stockholm, Sweden; 6 Department of Cardiology, Karolinska University Hospital, Stockholm, Sweden; 7 Department of Clinical Sciences Lund, Division of Infection Medicine, Lund University, Lund, Sweden; 8 Division for Infectious Diseases, Skåne University Hospital, Lund, Sweden

**Keywords:** *Staphylococcus aureus*, Bacteraemia, CIED, Management score, Endocarditis

## Abstract

Patients with cardiac implantable electronic device (CIED) and *Staphylococcus aureus* bacteraemia (SAB) are at risk of having infective endocarditis (IE). The objectives were to describe a Swedish population-based cohort of patients with CIED and SAB, to identify risk factors, and to construct a predictive score for IE. Patients over 18 years old in the Stockholm Region identified to have SAB in the Karolinska Laboratory database from January 2015 through December 2019 were matched to the Swedish Pacemaker and Implantable Cardioverter-Defibrillator ICD Registry to identify the study cohort. Data were collected from study of medical records. A cohort of 274 patients with CIED and SAB was identified and in 38 episodes (14%) IE were diagnosed, 19 with changes on the CIED, and 35 with changes on the left side of the heart. The risk factors predisposition for IE, community acquisition, embolization, time to positivity of blood cultures, and growth in blood culture after start of therapy in blood cultures were independently associated to IE. A score to identify patients with IE was constructed, the CTEPP score, and the chosen cut-off generated a sensitivity of 97%, specificity of 25%, and a negative predictive value of 98%. The score was externally validated in a population-based cohort of patients with CIED and SAB from another Swedish region. We found that 14% of patients with CIED and SAB had definite IE diagnosed. The CTEPP-score can be used to predict the risk of IE and, when negative, the risk is negligible.

**Supplementary information:**

The online version contains supplementary material available at 10.1007/s10096-023-04585-x.

## Introduction

Cardiac implantable electronic devices (CIEDs), including pacemaker (PM), implantable cardioverter defibrillator (ICD), cardiac resynchronization therapy-pacemaker (CRT-P), and cardiac resynchronization therapy-defibrillator (CRT-D), are used in a wide range of patients and are found in a growing cohort of patients [1]. When a patient with CIED has *Staphylococcus aureus* bacteraemia (SAB), the risk of CIED infection, pocket infection or infective endocarditis (IE), is high [2]. The diagnosis of CIED IE is based on positive blood cultures and visualization of vegetations or other structural changes indicating IE on echocardiography, transthoracic (TTE) or transoesophageal (TOE), positron emission tomography–computed tomography (PET-CT), or cardiac CT [2, 3]. In previous studies on the risk of IE in patients with SAB (Supplementary (S) Table 1) [4–7], CIED has been shown to be a risk factor [4, 6]. In patients with SAB and CIED, three risk factors, PM but not ICD, more than one device-related procedure, and growth in blood culture (BC) after start of therapy, have been found to predict IE [5] in a study from a tertiary referral centre.

The guidelines for treatment of CIED infections recommend extraction of the CIED in all cases of SAB due to the high risk of CIED infection [2], based on multiple studies, from tertiary centres, finding a high proportion of CIED infection in this category of patients (36–55%) [8–11]. However, the results from a recent population-based study showed that the rate of IE was only 19% and the risk of relapse in SAB was low despite short treatment times and that most patients were not subjected to CIED-extraction, indicating that missed CIED IE were few [12]. The authors suggested that in patients without pocket infection or changes on the CIED, extraction of the CIED might be omitted. Further, observations from studies have shown that patients with IE and CIED without changes on the CIED did not have the CIED extracted and was without relapse or other detrimental outcome [12, 13]. This implies that identification of patients with CIED changes is of interest. The discrepancy between the recommendations in the guidelines and the observations in these studies inspired us to further study patients with CIED and SAB.

The first aim was to describe a population-based cohort of patients with CIED and SAB, secondly to identify risk factors for CIED IE, and thirdly to develop a score predicting IE and to validate the score in an external cohort. Further, an aim was to evaluate risk factors for changes on the CIED. Finally, we wanted to suggest a management strategy for the patients.

## Materials and methods

### The cohort

The Swedish individual unique personal numbers were used to identify all consecutive patients with blood cultures (BC) positive for *S. aureus* from January 2015 to December 2019, obtained from the laboratory databases of Clinical Microbiology, Karolinska University Hospital, Stockholm, Sweden, with a catchment population of 1.9 million inhabitants. The patients’ identities were matched to the database of the Swedish Pacemaker and Implantable Cardioverter-Defibrillator ICD Registry (PMR), covering 97% of all pacemaker and ICD procedures in the Stockholm area [14]. Patients found in both registries were eligible to be included in the cohort. All patients with SAB older than 18 years with a CIED in place at the date of the positive BC constituted the study cohort. Data were collected by CC, validated by AB, and stored after ethical approval obtained from the Swedish Ethics Committee (2020–00314).

The validation cohort used in the study was the cohort from another region in Sweden, described in Berge et al. [12].

### Definitions

The definition of CIED infection and CIED IE was from Blomström-Lundqvist et al. [2]. All infections fulfilling the criteria for definite IE were referred to as CIED IE irrespective whether changes were found on the CIED or on the heart valves [2, 15]. Any significant changes, compatible with IE, seen on TTE or TOE, were considered to constitute a major structural criterion for CIED IE, and the changes seen in anatomical association to the CIED were described as CIED changes [16]. An episode of SAB was defined by the start of the clinical symptoms or signs in a patient resulting in BC taken showing growth of *S. aureus*. To discriminate BC taken within an episode from a recurrent infection, an episode was delimited by at least 14 days of effective treatment and clinical improvement. A later clinical condition resulting in a positive BC with growth of *S. aureus* within the study period was referred to as a “recurrent infection” or “recurrence.” Growth after start of therapy was defined to be positive if any BC showed growth *of S. aureus* after ≥ 24 h of therapy. Origin of infection and other focal infections caused by *S. aureus* were defined as described [17]. Briefly, to fulfil the criteria for diagnosing a focal infection, two out of three criteria had to be present, culture showing growth of *S. aureus* from the site of infection, signs or symptoms from the site of infection, and imaging results compatible with the diagnosis. Comorbidities were retrieved from the medical records prior to the episode and classified according to the Charlson index modified by Quan et al. [18, 19].

### Data collection and analysis

The collection of the microbiological data has been described (S material). Clinical data from each episode were collected from 365 days before the date of the first positive BC in the episode until 365 days after. The collected data from the patients’ medical records have been described (S material). The analysis of the collected data was conducted in Stata, version 15.1 (StataCorp, College Station, TX, USA). The odds ratios (OR) and their confidence intervals (CI) were calculated when applicable. The *χ*^*2*^-test was used when applicable, and otherwise, the *p*-value of Fisher’s exact test was used. Differences between continuous variables were analysed with Wilcoxon’s rank-sum test. Values have been presented as proportions in percent or medians with interquartile ranges (IQR). Multivariable logistic regression analyses (MVA) were done using forward stepwise regression. In some of the analyses, the “rule of ten” was disobeyed [20]. To be entered into the MVA, continuous variables, time to positivity in BC, age, and Charlson score, were dichotomized to best identify the outcome of the variables but prioritizing a high sensitivity. Dummy variables were created for different CIEDs and for place of acquisition. In the second MVA, a dummy variable was created for the non-nosocomial acquisition. Receiver operator characteristics (ROC) curves were constructed to identify the optimal cut off of both the dichotomized individual variables with continuous values and the scores. The area under the curve (AUC) and its confidence intervals were calculated. Variables significantly associated (*p* < 0.05) with the outcome in the univariable analysis were introduced into the MVAs, starting with the lowest *p*-value. Lack of data was replaced by zero in the data set, no other imputations were made, and no patients were lost in follow-up. The scores predicting IE in patients with SAB (PREDICT, PREDICT-SAB, VIRSTA, and POSITIVE) were calculated as described in the original publications [4–7], and the cut offs chosen by the authors were used.

## Results

### The cohort

**Fig. 1:**
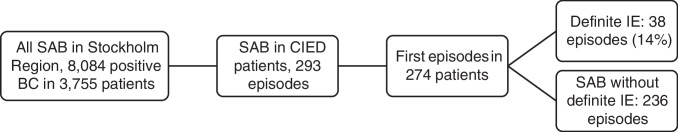
Flow chart of patients with CIED and SAB

The search in the laboratory databases resulted in 8084 BCs positive for *S. aureus* in 3755 patients from January 2015 to December 2019. The same patients appeared in both databases in 359 of the SAB episodes. In 66 of the episodes, no CIED was in place at the time of the positive BC. In 293 episodes in 274 patients, the criteria were fulfilled for inclusion in the study, having a CIED when SAB occurred (Fig. 1). The first episodes of SAB in a patient, 274 episodes, were further studied. Definite IE was diagnosed in 38 patients. In 19 patients, changes were seen on the CIED and changes were seen on the left side of the heart in 35 patients. No patient was diagnosed with definite IE without structural changes constituting a major criterion. Changes on the CIED were found in five patients not fulfilling the diagnostic criteria of definite CIED IE. The five patients had only one positive BC and had less than three minor criteria and thus not classified as definite IE. Thirty-eight patients had the CIED extracted, 19 diagnosed with definite CIED IE, and 19 without IE diagnosis (Table 1). Nineteen recurrent episodes were found in 16 patients, two in patients with IE, and in 14 patients without IE during the first episode.

**Table 1:** Clinical characteristics of the patients in the study. Univariate analysis of the difference between the subgroups diagnosed with definite CIED IE and without definite CIED IE

Characteristics	All (*n* = 274) (%)	Episodes with IE (*n* = 38) (%)	Episodes without IE (*n* = 236) (%)	Odds ratio (95% CI)	*P*-value
Age (years)	82 (74–87)	78 (68–86)	83 (75–87)	n/a	**0.013**
Sex (female)	74 (27)	12 (32)	62 (26)	1.3 (0.6–2.7)	0.49
Charlson score^2^	3 (2–4)	3 (2–4)	3 (2–4)	n/a	0.088
Charlson score ≥ 3^2^	169 (62)	20 (53)	149 (63)	0.65 (0.3–1.3)	0.22
Acquisition:					**0.033**
Community	47 (17)	12 (32)	35 (15)	2.7 (1.2–5.7)	**0.011**
Health care associated	126 (46)	17 (45)	109 (46)	0.9 (0.5–1.9)	0.87
Nosocomial	101 (37)	9 (23)	92 (39)	0.49 (0.22–1.1)	0.07
Present CIED not the first	99 (36)	17 (45)	82 (35)	1.5 (0.8–3.5)	0.23
CIED implantation (months)	32 (12–61)	29 (16–34)	32 (12–60)	n/a	0.81
Type of CIED:					0.53
PPM	193 (70)	25 (66)	168 (71)		
ICD	31 (11)	7 (18)	24 (10)		
CRT-P	21 (8)	2 (5)	19 (8)		
CRT-D	29 (11)	4 (11)	25 (11)		
Predisposition, any	59 (22)	13 (34)	46 (19)	2.1 (1.02–4.5)	**0.041**
Cardiac predisposition	57 (21)	13 (34)	44 (19)	2.3 (1.1–4.8)	**0.03**
Native valve disease	36 (13)	7 (18)	29 (12)	1.6 (0.7–4.0)	0.30
Prosthetic valve disease	37 (14)	7 (18)	30 (13)	1.6 (0.6–3.8)	0.34
Previous endocarditis	10 (4)	5 (13)	5 (2)	7 (1.9–25)	**0.001**
Intravenous drug user	3 (1.1)	1 (3)	2 (0.8)	3.1 (0.28–35)	0.36
Duration of symptoms (days)	1 (0.5–2)	1 (0.5–2)	1 (0.5–2)	n/a	0.32
Heart murmur	57 (21)	8 (21)	49 (21)	1.0 (0.44–2.3)	0.97
Fever ≥ 38 degrees	185 (68)	31 (82)	154 (65)	2.3 (1.0–5.6)	**0.046**
Embolization^3^	11 (4)	7 (18)	4 (2)	13 (3.6–47)	** < 0.001**
Sepsis or septic shock	75 (27)	13 (34)	62 (26)	1.5 (0.7–3.0)	0.31
Known origin of infection	97 (35)	9 (24)	88 (37)	0.5 (0.24–1.2)	0.10
Pocket infection	4 (1)	2 (5)	2 (1)	6.5 (0.9–48)	0.09
Wound infection	37 (14)	3 (8)	34 (14)	0.5 (0.1–1.7)	0.44
Catheter related infection	27 (10)	3 (10)	24 (10)	0.8 (0.2–2.6)	1.0
Spondylodiscitis	18 (7)	3 (8)	15 (6)	1.3 (0.35–4.6)	0.72
Respiratory tract infection	17 (6)	0 (0)	17 (7)	n/a	0.14
Urinary tract infection	11 (4)	1 (3)	10 (4)	0.6 (0.1–5)	1.0
Unknown origin of infection	177 (65)	29 (76)	148 (63)	1.9 (0.9–4.2)	0.10
CRP at admission	156 (87–232)	177 (124–263)	150 (79–232)	n/a	0.064
BC major criterion for IE	189 (67)	36 (100)	153 (65)	9.7 (2.3–42)	** < 0.001**
MRSA	5 (2)	1 (3)	4 (2)	7.5 (0.2–14)	0.53
Time to positive BC (hours)^2^	11 (8–17)	9 (6–10)	12 (8–18)	n/a	** < 0.001**
Time to positivity, < 15 hours^2^	197 (72)	36 (95)	161 (68)	8.4 (2.0–36)	**0.001**
Pos. BC after start of therapy^1^					
(as proportion of all)	33 (12)	10 (26)	23 (10)	3.3 (1.4–7.7)	**0.004**
(as proportion of cultured (96 patients))	33 (34)	10 (48)	23 (30)	2.8 (0.8–5.8)	0.13
*Management*					
TTE performed	155 (51)	32 (84)	119 (50)	5.2 (2.1–13)	** < 0.001**
TOE performed	115 (42)	36 (95)	78 (33)	36 (8–152)	** < 0.001**
TTE or TOE performed	173 (63)	38 (100)	135 (57)	n/a	** < 0.001**
CIED changes	23 (18)	18 (47)	5 (2)	42 (14–123)	** < 0.001**
PET-CT performed	5 (2)	5 (13)	0 (0)	n/a	** < 0.001**
CIED changes	1 (0.4)	1 (3)	0 (0)	n/a	0.14
Extraction of CIED	38 (14)	19 (50)	19 (8)	11 (5.2–25)	** < 0.001**
Treatment, total, (days)	16 (10–32)	39 (29–44)	14 (10–25)	n/a	** < 0.001**
*Outcome*					
Recurrence in SAB	16 (6)	2 (5)	14 (6)	0.9 (0.2–4.0)	1.0
Death within 30 days	77 (28)	5 (13)	72 (31)	0.35 (0.1–0.92)	**0.03**
Death within 365 days	148 (54)	19 (50)	129 (54)	0.83 (0.4–1.6)	0.59

Values are given as numbers and proportions (%) and for continuous variables as medians and IQR. The *p*-value of differences in continuous variable were calculated with Wilcoxon’s rank sum test. In categorical variables, the differences were calculated with *χ*^2^ test when applicable and Fisher’s exact test in other cases. Differences with a *p*–value of < 0.05 are considered significant and are shown in bold

*n/a* not applicable

^1^All included, cultured after 24–96 h after start of therapy

^2^Continous variables have been dichotomized, both shown

^3^The most common embolizations were major arterial emboli (7), pulmonary emboli (2), and intracranial embolization (3)

### Comparison of clinical variables in patients with CIED IE and patients without CIED IE

Clinical variables in patients with and without CIED IE were analysed using univariate analysis. Significant differences were found in age, community acquisition, predisposition for IE, fever, embolization, growth in BCs fulfilling the major criterion for IE, time to positivity of BC, and growth in BC after start of therapy (Table 1). In 96 patients (35%), one or more BCs were taken after start of therapy. In the entire cohort, 115 patients (43%) had a TOE performed and 5 patients (2%) had a PET-CT. The median treatment time in the non-IE group was 14 days (10–25 days IQR). Mortality after 30 days was significantly lower in the IE group, 13%, compared to 31% in the non-IE group. After exclusion of patients not surviving the first 14 days from the start of the episode, no significant difference in mortality was seen (patients with IE, 8%, patients without IE, 9%, *p*-value 1.0, data not shown). The overall 365-day mortality was 54%, not significantly different between the groups (Table 1).

### Variables independently associated to IE, receiver operator characteristics, and the scores

**Fig. 2:**
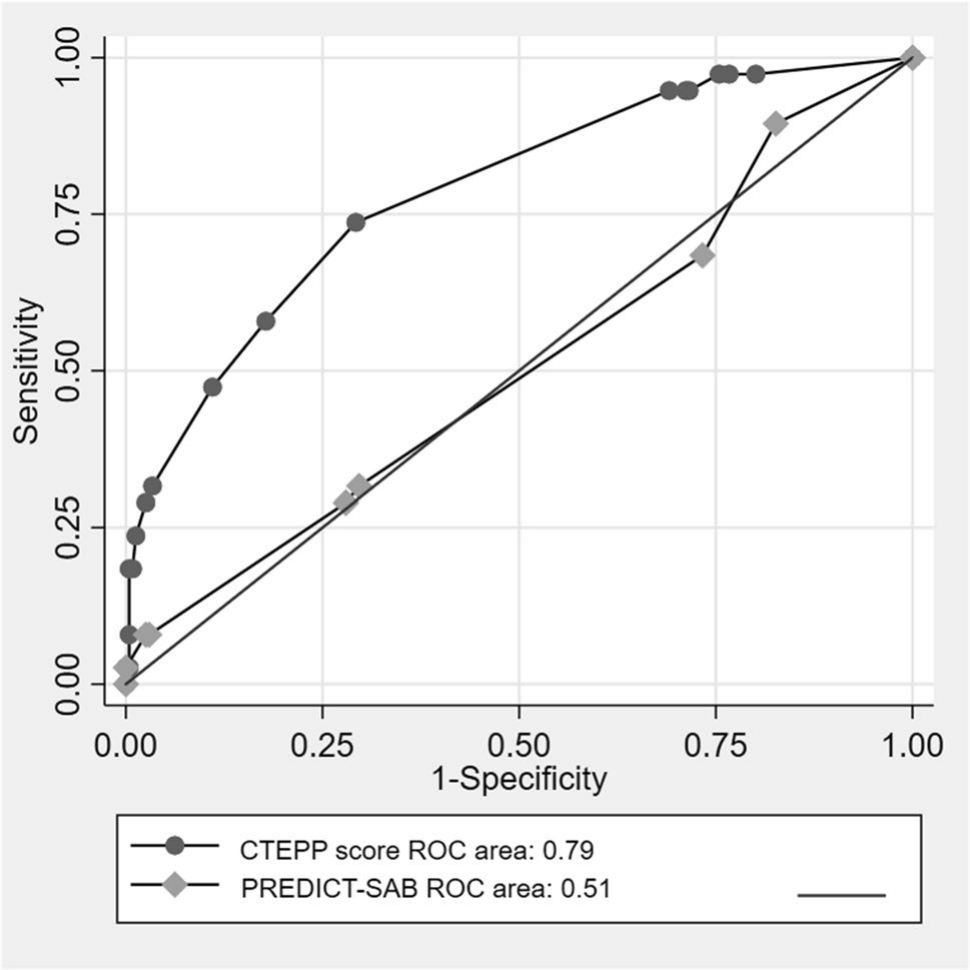
ROC curve of the correlation between the CTEPP score and CIED IE in patients with CIED and SAB. Comparison to the performance of the PREDICT-SAB score, also developed in a cohort of patients with CIED and SAB

Variables significantly correlated to IE in univariate analysis were introduced into the MVA. The lowest number of outcomes per variable in the analysis was six (38 outcomes and six variables). Predisposition for IE, community acquisition, embolization, time to positivity of the BC less than 15 h, and growth in BC after start of therapy were independently correlated to IE (Table 2). The likelihood ratio test showed that the model without restrictions best fitted the data (*p*-value 0.017). Based on the OR of the MVA, each variable was given a value to be used in a score (Table 2). The score was given the name the CTEPP score from the first letter of each variable (community acquisition, time to positivity, embolization, predisposition for IE, and positive in BC after start of therapy). Based on the sum of the value given each variable from the MVA, a ROC curve was plotted (Fig. 2). The AUC was 0.79 (CI 0.71–0.87), significantly higher than the PREDICT-SAB score (0.51 (CI 0.41–0.61)) (Fig. 2).

**Table 2:** Variables independently correlated to CIED IE in MVA by forward stepwise regression

Variables	Odds ratio (95% CI)	*p*-value	Suggested points in score
Predisposition for IE	2.4 (1.04–5.7)	0.040	1
Community acquisition	3.8 (1.6–9.3)	0.003	2
Embolization	12.6 (2.7–58)	0.001	6
Time to positivity ≤ 15 h	8.1 (1.8–36)	0.006	4
Positive BC after start of therapy	3.3 (1.2–6.7)	0.016	1.5

To optimize the sensitivity and negative predictive value, but with a specificity as high as possible, a cut off for a positive result of the score of ≥ 2 was chosen resulting in a sensitivity of 97%, specificity of 25%, a negative predictive value of 98%, and the ratio between true positive and total numbers of positive was six. Analysis was made of the performance of the scores developed to assess the risk of IE in SAB patients (Table 3). PREDICT day 2 [5] and the POSITIVE score [7] significantly predicted IE and had a sensitivity of 76% and 58%, respectively, to identify patients with IE (Table 3). The other scores did not identify patients with IE from patients without IE significantly better than chance.

**Table 3:** The scores, for evaluation the risk of IE of patients with SAB, were compared for the performance to identify patients with CIED IE in the cohort

Scores	IE (*n* = 38)	Non-IE (*n* = 236)	Odds ratio	*p*-value	Sens	Spec	PPV	NPV	AUC
PREDICT-SAB	4 (3.5–7.5)	4 (3.5–7.5)	n/a	0.85	n/a	n/a	n/a	n/a	0.51 (0.41–0.61)
Positive PREDICT score day 2	29 (76)	140 (59)	2.2 (1.0–4.9)	0.046	76 (60–89)	41 (34–47)	17 (12–24)	91 (84–96)	0.60 (0.51–0.70)
Positive PREDICT score day 5	38 (100)	236 (100)	0.16 (0.002–13)	1.0	100 (91–100)	0 (0–13)	14 (10–18)	n/a	0.65 (0.56–0.74)
Positive VIRSTA score (≥ 2 points)	38 (100)	236 (100)	0.16 (0.002–13)	1.0	100 (91–100)	0 (0–13)	14 (10–18)	n/a	0.69 (0.60–0.78)
Positive score (≥ 4 points)	22 (58)	98 (42)	1.9 (0.97–3.8)	0.06	58 (41–74)	58 (52–65)	18 (12–26)	90 (84–94)	0.71 (0.61–0.80)
Positive CTEPP score (≥ 2 points)	37 (97)	178 (75)	12 (1.6–90)	0.002	97 (86–100)	25 (19–31)	17 (12–23)	98 (91–100**)**	0.79 (0.71–0.87**)**

Comparison of the scores capacity to identify patients with CIED IE and without, based on the cut off specified on the publications, except for PRECIDT-SAB that does not have a specified cut off. The *p*-value of differences in the continuous variable were calculated with Wilcoxon’s rank sum test. In categorical variables, the differences were calculated with *χ*^2^ test. Proportions and confidence intervals in percent are shown in parenthesis

*Abbreviations*: *Sens* sensitivity, *Spec* specificity, *PPV* positive predictive value, *NPV* negative predictive value, *AUC* area under the curve, *n/a* not applicable

### The validation cohort

The performance of the CTEPP score, to identify IE in patients with CIED and SAB, was tested in a validation cohort, a population-based cohort of the same category of patients with CIED and SAB from another region in Sweden [12]. The cohort consisted of 177 episodes of SAB, and IE was diagnosed in 25 episodes. The sensitivity of the CTEPP score to identify patients with IE was 100%, the specificity 13%, and the negative predictive value was 100% (data not shown).

### Comparison of clinical variables between patients with CIED changes and patients without

To be able to identify risk factors associated with CIED changes found on any examination, a univariable analysis was performed on the Stockholm cohort using CIED changes as dependent variable. The analysis identified lower age, lower Charlson score, community and health care associated acquisition made into one variable, ICD, embolization, sepsis or septic shock at admission, pocket infection, short time to positivity of the BC, and growth in BC after start of therapy to be correlated to CIED changes (S Table 2).

In MVA, using forward stepwise regression, by first introducing the variable with the lowest *p*-value into the MVA, non-nosocomial acquisition (community and health care associated acquisition as one variable), ICD, embolization, and growth in BC after start of therapy were found to be independently correlated to CIED changes (Table 4). The likelihood ratio test showed that the model without restrictions best fitted the data (*p*-value < 0.001). The lowest number of outcomes per variable in the analysis was five (24 outcomes and five variables).

**Table 4:** Variables independently correlated to CIED changes by MVA

Variables	Odds ratio of the UVA (CI)	*p*-value of the UVA	Odds ratio of the MVA (CI)	*p*-value of the MVA
Acquisition:		**0.026**		
Community	2.2 (0.8–5.6)	0.15		
Health care associated	1.7 (0.7–4.0)	0.20		
Nosocomial	0.2 (0.06–0.76)	**0.01**		
Non-nosocomial^2^			7.5 (1.8–32)	**0.006**
Type of CIED:		0.53		
PPM	0.6 (0.3–1.7)	0.36		
ICD	3.7 (1.4–10)	**0.010**	5.4 (1.8–16)	**0.003**
CRT-P	n/a	0.23		
CRT-D	1.5 (0.4–5.5)	0.56		
Embolization	11 (3.0–38)	**0.001**	21 (4–119)	** < 0.001**
Pos. BC after start of therapy^1^	4.5 (1.8–12)	**0.003**	5.5 (1.8–16)	**0.002**

The odds ratios (OR) and their confidence intervals (CI) were calculated. MVA of the variables showing significant differences in univariate analysis was done using forward stepwise regression. Differences with a *p*-value of < 0.05 are considered significant and are shown in bold

^1^All included, cultured ≥ 24 h after start of therapy. ^2^Non-nosocomial was calculated as the sum of community and health care associated acquisition

## Discussion

In this study, we have described the clinical presentation of a population-based cohort of patients with CIED and SAB. Five variables, predisposition for IE, community acquisition, embolization, time to positivity in BCs less than 15 h, and growth in BCs after start of therapy, were identified as independent risk factors for IE and used in a score, the CTEPP score, with an excellent sensitivity to identify patients with CIED IE and with a high negative predictive value. We also found a low rate of CIED IE, 14%, in concordance with a recently published population-based report [12], but in contrast to previous reports [8–11], all from tertiary referral centres, that found much higher rates (36–55%).

Some studies during the last years have studied risk factors for IE in patients with SAB [4–7] but have identified different variables (S Table 1). Previously, only one study, the PREDICT-SAB study, from a tertiary referral centre and not population-based, has focused on risk factors for IE in patients with CIED [5], being the primary comparison to the results of this study. The study identified PM, more than one device-related procedure, and growth in BC after more than 3 days after start of therapy (given the description “prolonged SAB” and “SAB ≥ 4 days”) to be independently correlated to IE. Only growth in BC after start of therapy appeared in our study too, although defined differently. An explanation of the diverse findings could be the composition of the cohorts; our study being population-based making it more likely to be generalizable. Another striking difference between some other studies and our study is the rate of methicillin resistant *S. aureus* (MRSA) (40% and 2%, respectively) [5]. The rate of MRSA in our study was not different to the rate of MRSA in all BCs in the region. The difference in rate could likely be due to the selection of infections difficult to treat, caused by MRSA, in referral centres. All the studies have the weakness of being performed with retrospective data.

The international guidelines have recommended TOE to be performed in all cases of patients with CIED and SAB and to continue with further evaluation of the patient with repeated TOEs, PET-CT, or cardiac CT, if the suspicion of IE remains. In our study, the risk of CIED IE was low with a negative CTEPP score. We propose that in all patients with CIED and SAB, TOE should be performed, but also suggest that further evaluation could be omitted after both a negative TOE and a negative CTEPP score.

To address the question on how to identify patients with CIED changes, we have shown that some of the risk factors associated with CIED changes were the same and some differ from those we identify to be associated to IE (Tables 2 and 4), although the episodes overlap to a large extent in the present study (19 episodes of 43, 44%; Table 1). Community or health care associated acquisition, ICD, embolization, and growth in BCs after start of therapy were independently correlated to CIED changes. This indicates the biologically plausible hypothesis that risk factors differ between IE and CIED changes. We have refrained from presenting yet another score to predict CIED changes although it performed very well and could be valuable if extraction was to be performed only if CIED changes were found.

This study has several limitations. The first being the retrospective design limiting the acquisition of a full data set. In a limited proportion, 42% of the study cohort, TOE was performed, introducing the risk that some patients with CIED IE could have been misclassified. In the PREDICT-SAB study, 64% of the patients in the study population were examined with TOE [5]. The decision whether to perform a TOE in patient with SAB was decentralized to the treating doctors, their assessment of the risk of CIED IE, and the individual situation of the patient, probably explaining the low proportion of TOE performed. Further, in the group of patients that died within 14 days, the IE diagnosis was rare and can possibly contain misclassified cases. However, the median treatment time (14 days) and the limited number of relapses (6%) suggest that the number of misclassified patients was low. The MVA identified growth after start of therapy to be independently correlated to CIED IE, but only 35% of the patients were re-cultured. A prospective dataset would overcome these shortcomings.

The plethora of scores in patients with SAB can be confusing; therefore, a prospective multicentre study, collecting data from the identified risk factors in all the studies and other variables of interest, would have the possibility to illuminate this complicated clinical situation. A prospective validation study of three of the scores found the VIRSTA score to have the best performance [21] in a cohort of all patients with SAB. The study did not have the aim to identify previous known risk factors or new ones and the cohort only contained 11% CIED carriers. Further, both PREDICT-SAB and the present study indicated that the risk factor profile is different between SAB episodes in patients with CIED and SAB episodes in patients without CIED. Finally, identification of patients with and without CIED IE and with and without changes on the actual leads, CIED changes, may be the foundation for exciting studies on in what situations extraction of the CIED should be recommended. Thus, the results of prospective multicentre studies of CIED and SAB cohorts would be most interesting. Waiting for those studies, we think that the clinician can be helped by the results of this study, the largest study on risk factors for IE in patients with CIED and SAB so far, to assess the risk CIED IE.

## Supplementary information

Below is the link to the electronic supplementary material.

Supplementary file1 (DOCX 49 KB)

## Data Availability

The datasets analysed during the current study are available from the corresponding author on reasonable request.
