# Supplementary material for: Staphylococcus aureus bacteraemia, cardiac implantable electronic device, and the risk of endocarditis: a retrospective population–based cohort study
Source: Eur J Clin Microbiol Infect Dis. 2023 Mar 15;42(5):583–91. doi: 10.1007/s10096-023-04585-x (PMC10105663; doi:10.1007/s10096-023-04585-x)
Supplement: Supplementary file 1 — Supplementary file1 (DOCX 49 KB) [file 10096_2023_4585_MOESM1_ESM.docx]

Supplementary tables

S table 1. Schematic representation of the scores to evaluate patients with SAB and the different risk factors used in each score to evaluate the risk of IE.

| Risk factor for IE | PREDICT-SAB | PREDICT day 2 | PREDICT day 5 | VIRSTA | POSITIVE | CTEPP |
| --- | --- | --- | --- | --- | --- | --- |
| Present CIED not the first | X |  |  |  |  |  |
| PPM | X | X | X | X |  |  |
| ICD |  | X | X | X |  |  |
| Positive BC after start of therapy | X |  | X | X |  | X |
| Time to positivity in BC |  |  |  |  | X | X |
| Community acquisition (and HCA) |  | X | X | X |  | X |
| Embolization |  |  |  | X |  | X |
| Predisposition |  |  |  | X | X | X |
| CRP > 190 |  |  |  | X |  |  |
| Iv drug use |  |  |  | X | X |  |
| Sepsis |  |  |  | X |  |  |
| Osteomyelitis |  |  |  | X |  |  |
| Meningitis |  |  |  | X |  |  |

Footnote: variables found to correlate to IE in the studies (X).

S table 2. Clinical characteristics of the patients in the study. Univariate analysis of the difference between the episodes where changes on the CIED were found and episodes without.

| **Characteristics** | **All**  **(n=274)** | **Episodes with CIED changes**  **(n=24)** | **Episodes without CIED changes**  **(n=250)** | **Odds ratio**  **(95% CI)** | ***P*-value** |
| --- | --- | --- | --- | --- | --- |
| Age (years) | 82 (74-87) | 72 (64-80) | 83 (76-87) | n/a | **0.001** |
| Sex (female) | 74 (27) | 5 (21) | 69 (28) | 0.7 (0.4-1.9) | 0.48 |
| Charlson score | 3 (2-4) | 2 (0-4) | 3 (2-4) | n/a | **0.020** |
| Acquisition: |  |  |  |  | **0.026** |
| Community | 47 (17) | 7 (29) | 40 (16) | 2.2 (0.8-5.6) | 0.15 |
| Health care associated | 126 (46) | 14 (58) | 112 (45) | 1.7 (0.7-4.0) | 0.20 |
| Nosocomial | 101 (37) | 3 (12) | 98 (39) | 0.2 (0.06-0.76) | **0.01** |
| Present CIED not the first | 99 (36) | 7 (29) | 92 (37) | 0.7 (0.3-1.8) | 0.46 |
| CIED implantation (months) | 32 (12-61) | 26 (11-65) | 32 (12-60) | n/a | 0.90 |
| Type of CIED: |  |  |  |  | 0.53 |
| PPM | 193 (70) | 14 (58) | 179 (72) | 0.6 (0.3-1.7) | 0.36 |
| ICD | 31 (11) | 7 (29) | 24 (10) | 3.7 (1.4-10) | **0.010** |
| CRT-P | 21 (8) | 0 (0) | 21 (8) | n/a | 0.23 |
| CRT-D | 29 (11) | 3 (12) | 26 (10) | 1.5 (0.4-5.5) | 0.56 |
| Predisposition, any | 59 (22) | 3 (12) | 56 (22) | 0.49 (0.14-1.7) | 0.26 |
| Cardiac predisposition | 57 (21) | 3 (12) | 54 (22) | 0.5 (0.15-1.8) | 0.29 |
| Native valve disease | 36 (13) | 1 (4) | 35 (14) | 0.3 (0.03-2.0) | 0.34 |
| Prosthetic valve disease | 37 (14) | 3 (12) | 34 (14) | 0.9 (0.3-3.2) | 1.0 |
| Previous endocarditis | 10 (4) | 2 (8) | 8 (3) | 2.8 (0.5-14) | 0.22 |
| Intravenous drug user | 3 (1) | 1 (4) | 2 (1) | 5.4 (0.5-62) | 0.24 |
| Duration of symptoms (days) | 1 (0.5-2) | 1.25 (0.75-2) | 1 (0.5-2) | n/a | 0.071 |
| Heart murmur | 57 (21) | 3 (12) | 54 (22) | 0.5 (0.15-1.8) | 0.29 |
| Fever ≥38 degrees | 185 (68) | 17 (71) | 168 (67) | 1.2 (0.5-3.0) | 0.72 |
| Embolization | 11 (4) | 5 (21) | 6 (2) | 11 (3.0-38) | **0.001** |
| Sepsis or septic shock | 75 (27) | 11 (46) | 64 (26) | 2.5 (1.05-5.8) | **0.034** |
| Known origin of infection | 97 (35) | 8 (33) | 89 (36) | 0.5 (0.4-2.2) | 0.82 |
| Pocket infection | 4 (1) | 2 (8) | 2 (1) | 11 (1.5-84) | **0.040** |
| Unknown origin of infection | 177 (65%) | 16 (67) | 161 (64) | 1.1 (0.5-2.7) | 0.82 |
| CRP | 156 (87-232) | 195 (106-274) | 153 (81-230) | n/a | 0.19 |
| BC major criterion for IE | 189 (67) | 19 (79) | 170 (68) | 1.8 (0.6-5.0) | 0.26 |
| Time to positive BC (hours) | 11 (8-17) | 9 (6-12) | 12 (8-17) | n/a | **0.028** |
| Pos. BC after start of therapy |  |  |  |  |  |
| (all) | 33 (12) | 8 (25) | 25 (10) | 4.5 (1.8-12) | **0.003** |
| (only cultured, 95 patients) | 32 (34) | 8 (53) | 24 (30) | 2.7 (0.87-8.2) | 0.08 |
| Positive CTEPP score (≥2 p) | 215 (78) | 22 (92) | 177 (77) | 3.2 (0.7-14) | 0.10 |
| Positive CTEPP score (≥4 p) | 199 (73) | 22 (92) | 177 (71) | 4.5 (1.04-20) | **0.029** |
| *Management* |  |  |  |  |  |
| TEE performed | 115 (42) | 24 (100) | 91 (36) | 36 (8-152) | **<0.001** |
| TTE or TEE performed | 173 (63) | 24 (100) | 149 (60) | n/a | **<0.001** |
| Changes on CIED | 23 (8) | 23 (96) | 0 (0) | n/a | **<0.001** |
| PET-CT performed | 5 (2) | 3 (12) | 2 (1) | 18 (3-112) | **0.005** |
| Changes on CIED | 1 (0.4) | 1 (4) | 0 (0) | n/a | 0.09 |
| Extraction of CIED | 38 (14) | 18 (75) | 20 (8) | 34 (12-97) | **<0.001** |
| Treatment, total, (days) | 16 (10-32) | 34 (29-44) | 15 (10-29) | n/a | **<0.001** |
| *Outcome* |  |  |  |  |  |
| Definite IE | 38 (14) | 19 (79) | 19 (8) | 46 (15-137) | <**0.001** |
| Recurrence in SAB | 16 (6) | 2 (8) | 14 (6) | 1.5 (0.3-7.2) | 0.64 |
| Death within 30 days | 77 (28) | 2 (8) | 75 (30) | 0.2 (0.05-0.92) | **0.024** |
| Death within 365 days | 148 (54) | 10 (42) | 138 (55) | 0.58 (0.2-1.4) | 0.20 |

Footnote: Values are given as numbers and proportions (%) and for continuous variables as medians and IQR. The *p*-value of differences in continuous variable were calculated with Wilcoxon’s rank sum test. In categorical variables, the differences were calculated with χ^2^ test when applicable and Fisher’s exact test in other cases. Differences with a *p*–value of <0.05 are considered significant and are shown in bold.
